# Casting of a superhydrophobic membrane composed of polysulfone/Cera flava for improved desalination using a membrane distillation process

**DOI:** 10.1039/c7ra12474k

**Published:** 2018-01-08

**Authors:** Saikat Sinha Ray, Shiao-Shing Chen, Cao Thanh Ngoc Dan, Hung-Te Hsu, Hau-Ming Chang, Nguyen Cong Nguyen, Hau-Thi Nguyen

**Affiliations:** Institute of Environmental Engineering and Management, National Taipei University of Technology No. 1, Sec. 3, Zhongxiao E. Rd. Taipei-10608 Taiwan f10919@ntut.edu.tw; Faculty of Environment and Natural Resources, DaLat University Viet Nam

## Abstract

Superhydrophobic membranes are necessary for effective membrane-based seawater desalination. This paper presents the successful fabrication of a novel electrospun nanofibrous membrane composed of polysulfone and Cera flava, which represents a novel class of enhanced performance membranes consisting of a superhydrophobic nanofibrous layer and hydrophobic polypropylene (PP). Cera flava, which helps lower the surface energy, was found to be the ideal additive for increasing the hydrophobicity of the polysulfone (PSF) polymeric solution because of its components such as long-chain hydrocarbons, free acids, esters, and internal chain methylene carbons. In the fabricated membrane, consisting of 10 v/v% Cera flava, the top PSF–CF nanofibrous layer is active and the lower PP layer is supportive. The hybrid membrane possesses superhydrophobicity, with an average contact angle of approximately 162°, and showed high performance in terms of rejection and water flux. This work also examined the surface area, pore size distribution, fiber diameter, surface roughness, mechanical strength, water flux, and rejection percentage of the membrane. The salt rejection was above 99.8%, and a high permeate flux of approximately 6.4 LMH was maintained for 16 h of operation.

## Abstract 2

Superhydrophobic membranes for effective MD desalination.

## Graphical abstract

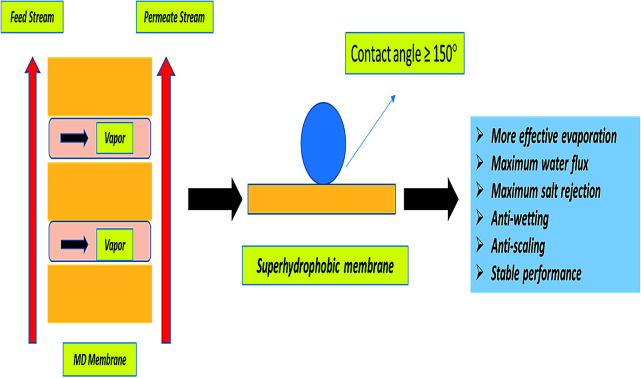

## 1. Introduction

A clean water supply is crucial for various aspects of modern life, such as public health, agriculture, and industrial production. However, the scarcity of clean water is a serious concern for many countries. The increasing scarcity of water is also a result of heightened water demand for households, industry, and agriculture. To solve this problem, effective and low-cost techniques for decontaminating water are being investigated.^1^ Currently, many advanced technologies based on membranes have been developed for water reuse and desalination. Reverse osmosis is a well-known methodology, but it is considerably more expensive than other available technologies; moreover, its use is constrained by the limited amount of permeate water that can be recovered and the environmental impact of the toxic residue remaining after the process. Therefore, a cheaper and more sustainable process with a high recovery rate for water reclamation is required.^2^

The membranes used in membrane distillation (MD) processes, which are commonly composed of hydrophobic materials such as polypropylene (PP), polytetrafluoroethylene (PTFE), and polyvinylidene fluoride (PVDF), are generally fabricated by processes such as thermally induced phase separation, stretching, and phase inversion.^3^ Direct contact membrane distillation (DCMD) configuration is a type of MD where an aqueous solution at a lower temperature is in direct contact with the permeate stream of the membranes. DCMD has been widely studied because of its convenience and simplicity.^4^ Recently, the electrospinning technique has been widely used to cast highly hydrophobic MD membranes. Electrospinning is a versatile technology for casting nonwoven sheets of nanofibrous polymeric material for membrane-based purification and separation.^5^ The electrospun nanofibrous material can easily be spun into structures possessing small pore size and a high surface-area-to-volume ratio, which can be further utilized for MD processes offering high water flux and salt or solute rejection.

Currently, superhydrophobic-surface-specialized structures with versatile characteristics have attracted broad attention in the field of desalination as well as absorption and filtration. Superhydrophobic specialized surfaces fabricated by electrospinning processes have several advantages such as high porosity, submicron pore diameter, nanoscale rough surface morphology, high permeability, and a large surface-area-to-volume ratio.^6^ MD processes should be able to continuously generate pure water with lower conductivity. However, membrane pore wetting and liquid penetration during long-term MD operation results in decreased salt rejection. Recently, some researchers have shown that an increase in the hydrophobicity of MD membranes can decrease pore wetting effectively.^7^ Therefore, some researchers are focusing on the development of superhydrophobic membranes by producing rough surfaces or utilizing hydrophobic additives.^8^ Superhydrophobicity introduces an air gap between water and the membrane surface, which can potentially increase the allowable pore sizes before membrane pore wetting takes place, resulting in higher mass flux.^9^ Additionally, superhydrophobic membranes limit the heat loss due to conduction, which would otherwise decrease the evaporation (which works as a driving force) and compromise vapor flux. Thus, superhydrophobic modification could decrease the heat loss by conduction across the membrane.^10^

**Fig. 1:**
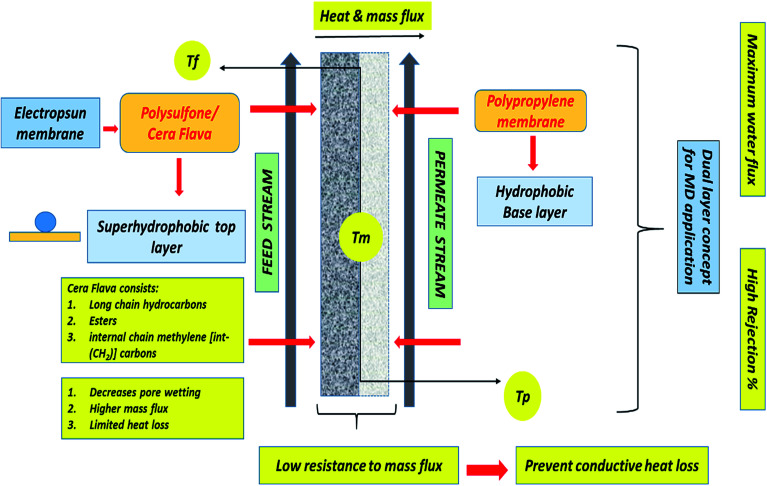
Surface modification of a polypropylene membrane with an electrospun nanofibrous polysulfone layer incorporating Cera flava for improved MD applications.

In this study, for the first time, Cera flava has been incorporated into polysulfone (PSF) solution to modify the PP membrane for MD application. Cera flava consists of various components that are typically hydrophobic in nature, such as long-chain hydrocarbons, free acids, and esters. In addition, most of the carbons in Cera flava are internal chain methylene [int-(CH_2_)] carbons; this specialized structure is responsible for its superhydrophobicity. Cera flava is generally a waste product from honey-based industries. Therefore, it is an economical and easily available material categorized as a solid lipid with very low surface energy; that is, it comprises physiological and compatible lipids with a very high melting point as the solid core. The hydrophobicity of the PSF solution increases with increasing Cera flava concentration. Previous studies have successfully increased surface roughness, resulting in increased surface hydrophobicity of PP membranes, by utilizing the electrospinning of PSF and incorporating Cera flava solution on the PP surface.^6,11^ Finally, sodium dodecyl sulfate (SDS) surfactant was doped with polysulfone (PSF) for uniformity and homogeneity in fiber diameter. This anionic surfactant was found to be the ideal additive for increasing the conductivity of the PSF polymeric solution, which helps in lowering the critical voltage required to start the electrospinning process, resulting in greater elongation of the nanofibers because of the increase in charge density.^12^Fig. 1 illustrates the detailed mechanism of the surface modification of the PP membrane by PSF–CF. The figure also clarifies why the superhydrophobic PSF–CF layer was placed on the feed solution side.

For instance, electrospun membranes have been reported in many studies and have shown very interesting outcomes while utilizing in MD application. A brief summary of different electrospun nanofibrous membranes utilized in MD system is shown in Table 1. The properties of the proposed membrane were compared with that of other electrospun nanofibrous membranes based on pore size range, contact angle, porosity and membrane material. The present composite membrane achieved higher contact angle with higher degree of porosity.

**Table 1:** A brief overview of various electrospun membranes synthesized for MD application

Technique utilized	Membrane material	Porosity	Pore size range	Average contact angle	References
Electrospinning with post treatment	PVDF	80%	0.18 μm	≥150°	13
Electrospinning followed by hot pressing	PVDF–HFP	58%	0.26 μm	125°	14
Electro-spinning followed by sintering	PTFE	72–82%	—	136.1–157.3°	15
Electrospinning	PVDF	58.87%	1.0 μm	143–147°	16
Electrospinning with post heat treatment	PSF–CF/PP	80–81%	0.1 μm	150–162°	Present study

With the current developments in MD technology, recent research has focused on improving membranes by modifying or changing the surface chemistry.^17^ This paper includes factors such as high salt rejection (>99%) and high-water flux due to a high surface-area-to-pore-volume ratio; hence, it also confirms the feasibility of the proposed surface-modified membrane. These features place the PSF–CF/PP in a novel category of bilayered membranes for MD processes. Finally, the overall performance of the modified membrane has been thoroughly examined using a thermally driven MD operation to evaluate the water flux and salt rejection of a 30 g L^−1^ NaCl aqueous solution. Moreover, this paper provides the fundamental concepts for developing other novel bilayered superhydrophobic membranes for MD processes.

## 2. Materials and methods

### 2.1 Starting material

PSF with a molecular weight of 35 000 was ordered from Sigma Aldrich by LS, sodium dodecyl sulfate (SDS) was purchased from Sigma Aldrich and natural honey was acquired from a forest in Taiwan. PP was provided by BenQ, Taiwan.

### 2.2 Experimental

#### 2.2.1 Preparation of PSF solution for electrospinning

First, PSF pellets were dissolved in a dimethylformamide solution at a concentration of 16% w/v% inside a water bath at 70–80 °C under constant stirring with a magnetic stirrer at 700–800 rpm. A thick solution was obtained after the aforementioned conditions were maintained for 3–4 h without disruption. Finally, 1% w/v of SDS (sodium dodecyl sulfate) was incorporated in the above solution for production of uniform nanofibers.

#### 2.2.2 Extraction of Cera flava from natural honey

Cera flava is a component of natural honey, which can be easily separated by heating the honey at 110–120 °C inside a water bath; Cera flava forms a yellow layer on top that can be separated using a spatula. Subsequently, in our experiment, 10% w/v of Cera flava was dissolved in dimethylformamide at 50–60 °C under constant stirring with a magnetic stirrer at 300–400 rpm for 2 h to avoid precipitation.

#### 2.2.3 Incorporation of Cera flava into PSF solution

The solution of PSF and Cera flava was cooled to room temperature (25 °C), and Cera flava at 5%, 7.5%, and 10% (v/v) was added to the 16% (w/v) PSF solution. The solution was stirred vigorously for 30 min with a magnetic stirrer at room temperature (25 °C).

#### 2.2.4 Electrospinning processing parameters

**Fig. 2:**
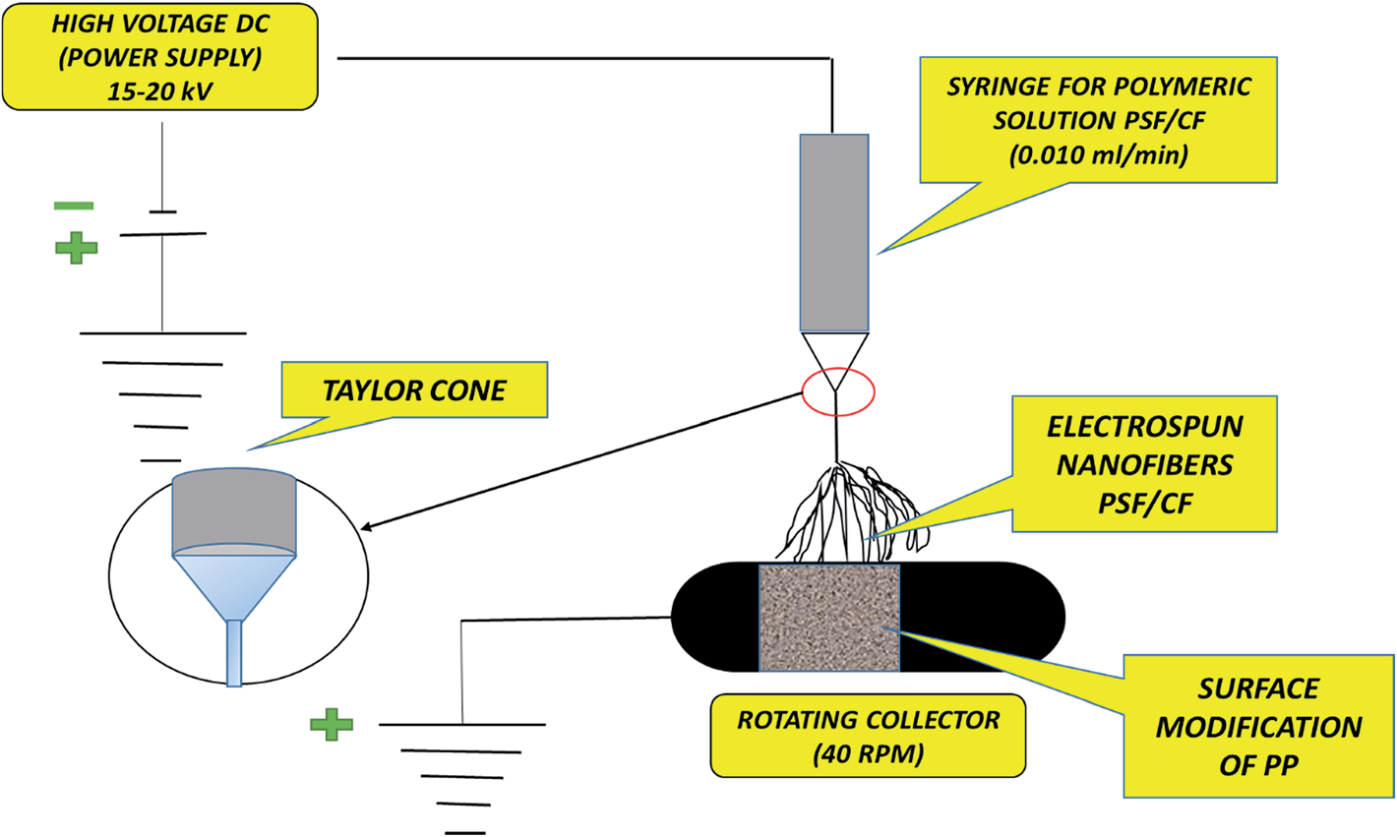
Typical block diagram of the electrospinning technique.

The electrospinning instrument used in this study was purchased from Falco Tech Enterprise Co., Ltd., Taiwan, and Fig. 2 presents a block diagram of the electrospinning technique. The setup consisted of three components: (1) a voltage supplier, (2) a syringe/capillary tube with needles, and (3) a collecting screen/roller.^18^

Table 2 lists the electrospinning process parameters such as the voltage, flow rate, current temperature, and distance between the needle tip (jet) and metal collector (roller) applied in the electrospinning of the PSF–CF solution at various concentrations onto the PP mats.

**Table 2:** Process parameters used for surface modification of the PP membrane with PSF–CF

Membrane type (concentration % of electrospinning solution)	Temperature (°C)	Flow rate (ml min^−1^)	Applied voltage (kV)	Current (mA)	Distance between needle tip (jet) and metal collector (cm)	Metal Collector Roller (rpm)
16% PSF–CF_(5%)_/PP	24.5	0.008	19	0.004	12	30
16% PSF–CF_(7.5%)_/PP	24.5	0.008	19	0.004	12	30
16% PSF–CF_(10%)_/PP	24.5	0.008	19	0.004	12	30

#### 2.2.5 Heat-pressing posttreatment

To increase the membrane stability and mechanical durability, heat-pressing posttreatment was performed by pressing the nanofibrous layer (PSF–CF) and PP between two flat irons heated in an oven at approximately 120 °C for 2 h. This process has been discussed in many recent studies.^19^

#### 2.2.6 Membrane distillation

**Fig. 3:**
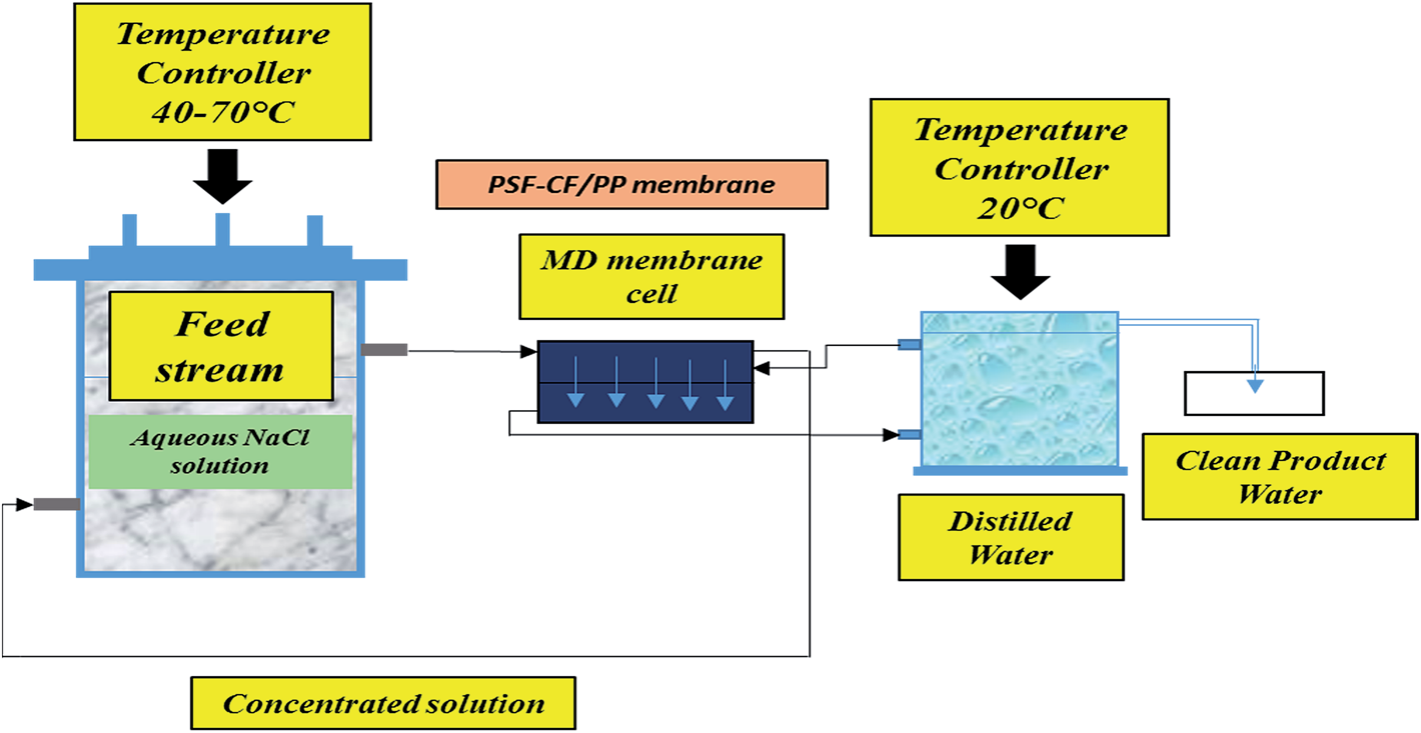
Schematic of a typical setup for lab DC-MD.

Lab-scale MD was performed as shown in Fig. 3. The DC-MD experimental test cell equipment was acquired from Sterlitech Corporation Ltd. (USA). Observations were thoroughly evaluated for multiple temperatures and durations. The schematics of a typical setup for lab MD are presented in Fig. 3.

The permeate flux *J*_w_ (L m^−2^ h^−1^; LMH) was measured in terms of the total volume of the permeate water using eqn (1)^2,20^1
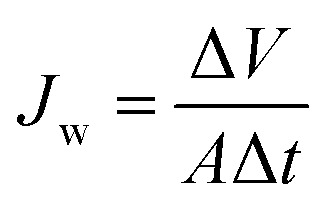
where *V* is the total volume of the permeate over a particular period Δ*t* (h) and *A* is the surface area of the membrane used in the MD system. The salt rejection of the system, indicated as the percentage of NaCl retained by the membrane, was calculated using eqn (2).^2^2
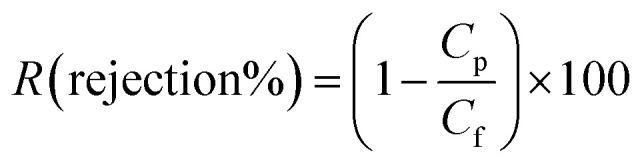
where *C*_p_ (g L^−1^) is the solute concentration in the product stream and *C*_f_ (g L^−1^) is the solute concentration in the feed stream.

### 2.3 Characterization

The surface morphology was studied using scanning electron microscopy (SEM; JEOL JSM-5900, Japan). The contact angle of the membrane was analyzed with the OSATM optical surface analyzer (OSA60-G, Ningbo NB Scientific Instruments Co., Ltd., China) to measure its hydrophobicity. The surface roughness was examined with atomic force microscopy (AFM). Membrane surface properties such as pore size, pore diameter, and surface area were observed using an adsorption/desorption analyzer (Micromeritics N_2_, ASAP 2020, USA).

### 2.4 Quality control

To obtain reliable results, the experiments were performed in triplicate and the mean values were recorded. Error bars are based on the standard errors of the three replicate tests.^21^

## 3. Results and discussion

### 3.1 Physical properties of solution

#### 3.1.1 Conductivity

The solvent used to dissolve the polymer must possess some degree of higher conductivity; alternately, organic or inorganic additives can be utilized to spike the conductivity of the polymeric solution in order to decrease the critical voltage required to initiate the electrospinning process. Increasing the conductivity of the solution also improves the quality of the fibers, because the higher level of charges carried by the solution increases the stretching of the polymeric solution from the jet.^18*a*,22^ SDS surfactant being anionic in nature was found to be the ideal additive for increasing the conductivity of the polymeric solution, which helps in lowering the critical voltage required to start the electrospinning process. Whereas, Cera flava is an ideal additive because in addition to promoting superhydrophobicity, the conductivity is also increased. Table 3 shows that the conductivity of PSF solution is increased hundred times by the addition of SDS and Cera flava. The quality of the nanofibers, as analyzed by SEM micrographs, is shown in the next section.

**Table 3:** Physical properties of polymeric solution used in electrospinning [note: 16% w/v of PSF and 1% w/v SDS was used in all cases except the first one]

Polymeric solution	Conductivity (μS cm^−1^)	Viscosity (cP)
PSF (only)	10.5	121.3
PSF–CF_(0%)_	1105.5	135.0
PSF–CF_(5%)_	1157.1	135.9
PSF–CF_(7.5)_	1182.0	137.6
PSF–CF_(10%)_	1197.9	139.1

#### 3.1.2 Viscosity

Typically, for a highly viscous polymer solution, the electrical charges cannot produce sufficient force to stretch the polymeric solution to form fibers. Below a certain concentration, the electrospinning jet may even break up into tiny droplets such that fibers do not form. A higher molecular weight polymer or higher polymeric solution concentration results in increased viscosity.^23^ However, the polymeric solution must possess optimum viscosity for more effective processing of the electrospinning technique. Table 3 indicates how the viscosity changes with the increased concentration of Cera flava in the PSF solution, although the increase is insubstantial.

### 3.2 Characterization of the membranes

#### 3.2.1 Contact angle analysis

**Fig. 4:**
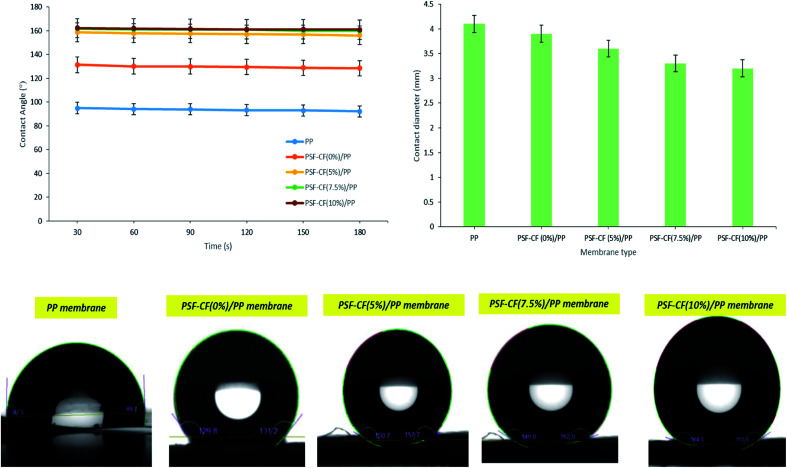
Contact angle and contact diameter analysis of different membranes [note: error bars are based on standard errors from three replicate tests].

The contact angle of the PP membrane and modified membranes (PSF–CF/PP) were analyzed by the OSATM optical surface analyzer (OSA60-G, Ningbo NB Scientific Instruments Co., Ltd., China). In general, the contact angle indicates the nature of the membrane surface with regard to hydrophilicity or hydrophobicity. The polypropylene membrane was found to be hydrophobic. After the surface modification of the PP membrane with PSF–CF, the contact angle indicated superhydrophobicity. Interestingly, the average contact angle of the modified surface increases as the concentration of PSF–CF increases from 5% to 10%. Fig. 4 indicates the contact angle analysis along with the nature of the membrane surface utilized in the MD process. The contact diameter was also measured, and the data indicates that a greater contact angle is correlated with a lower contact diameter. Thus, PSF–CF_(10%)_/PP shows the highest contact angle (162°) and the lowest contact diameter (3.1 mm).

#### 3.2.2 FT-IR analysis

**Fig. 5:**
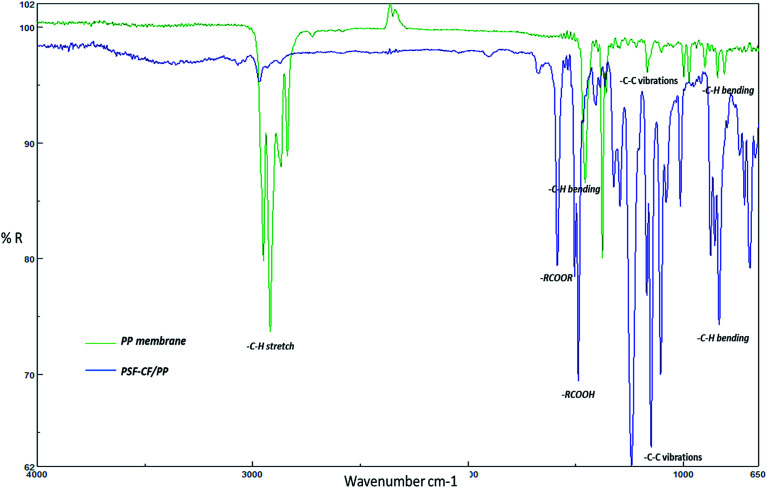
FT-IR analysis of the PP membrane and surface-modified PSF–CF/PP membrane [note: FT-IR analysis was carried out in the range of 600–4000 cm^−1^].

FT-IR spectra were recorded using an instrument acquired from PerkinElmer (Waltham, MA, USA). The FT-IR analysis was carried out in the range of 600–4000 cm^−1^ to understand and confirm the presence of all desired functional groups in the PSF–CF nanofibrous and PP membranes. As Fig. 5 indicates, strong peaks were observed at the following ranges: 2950–2850 cm^−1^ (C–H stretching), 1450–1500 cm^−1^ (–C–H bending), 1300–1250 cm^−1^ (–C–C vibrations), and 860–680 cm^−1^ (–C–H bending). It was observed that, more sharp peaks of hydrocarbon were obtained in case of PSF–CF/PP. These hydrocarbons indicate the hydrophobicity of the fabricated membrane. Beside that, a sharp peak of ester functional group was observed in PSF–C/PP that confirms the presence of Cera flava in the casted membrane. Thus, it can be concluded that presence of hydrocarbons and ester functional groups are responsible for superhydrophobicity of PSF–CF/PP membrane.

#### 3.2.3 SEM analysis

**Fig. 6:**
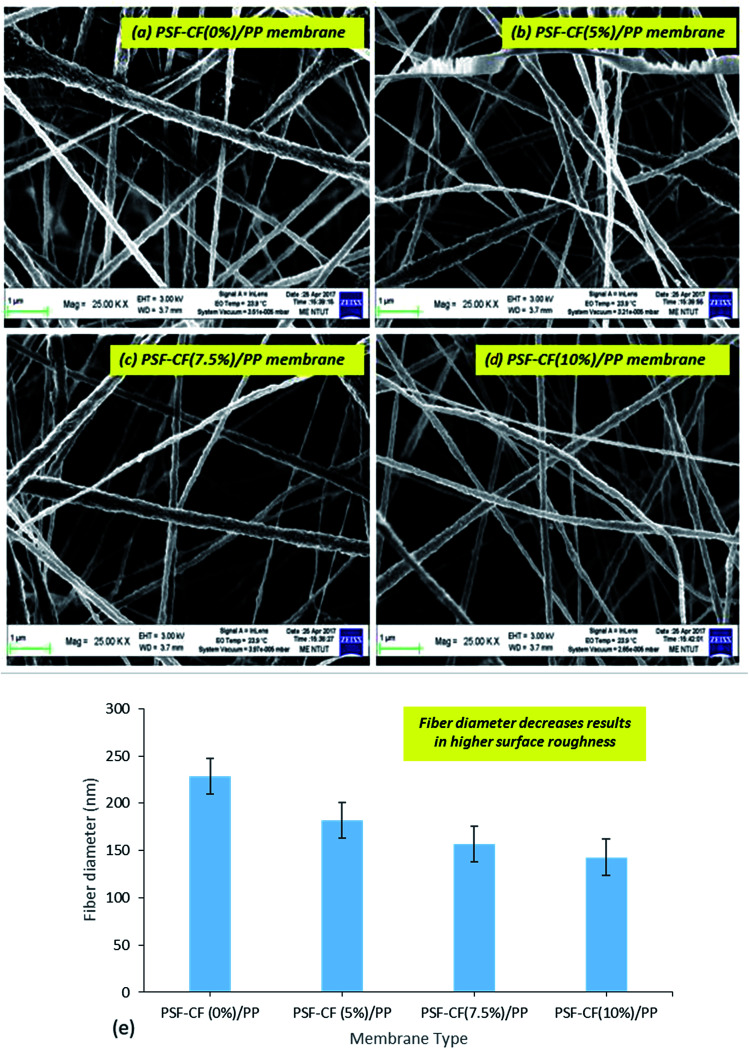
SEM image analysis: (a) 16% PSF (b) 16% PSF–CF_(5%)_ (c) 16% PSF–CF_(7.5%)_; (d) 16% PSF–CF_(10%)_ (e) fiber diameter analysis for different concentrations of PSF incorporated with CF [note: error bars are based on standard errors by analyzing at least 10 measurements].

The morphology of electrospun nanofibrous membranes was analyzed using field emission SEM (JOEL, JSM 7600 F, Japan). The PSF–CF nanofibrous layer must be placed on top to avoid them being covered by the supportive layer of the PP membrane. Fig. 6 shows a detailed morphological analysis, presenting multiple SEM micrographs to demonstrate the effect of including Cera flava in the PSF nanofibers. In addition, the fiber diameter was analyzed from the SEM micrographs by image analysis software (Image J2X). Interestingly, the fiber diameter decreased slightly with the increasing concentration of PSF–CF, resulting in greater surface roughness. Typically, a more conductive polymeric solution will produce greater stretching of the electrospinning jet due to the presence of more charge carriers that has been supported by Table 2. This would favour a reduction in fiber diameter. These observations support that the incorporation of Cera flava in the PSF solution reduces fiber diameter.

**Fig. 7:**
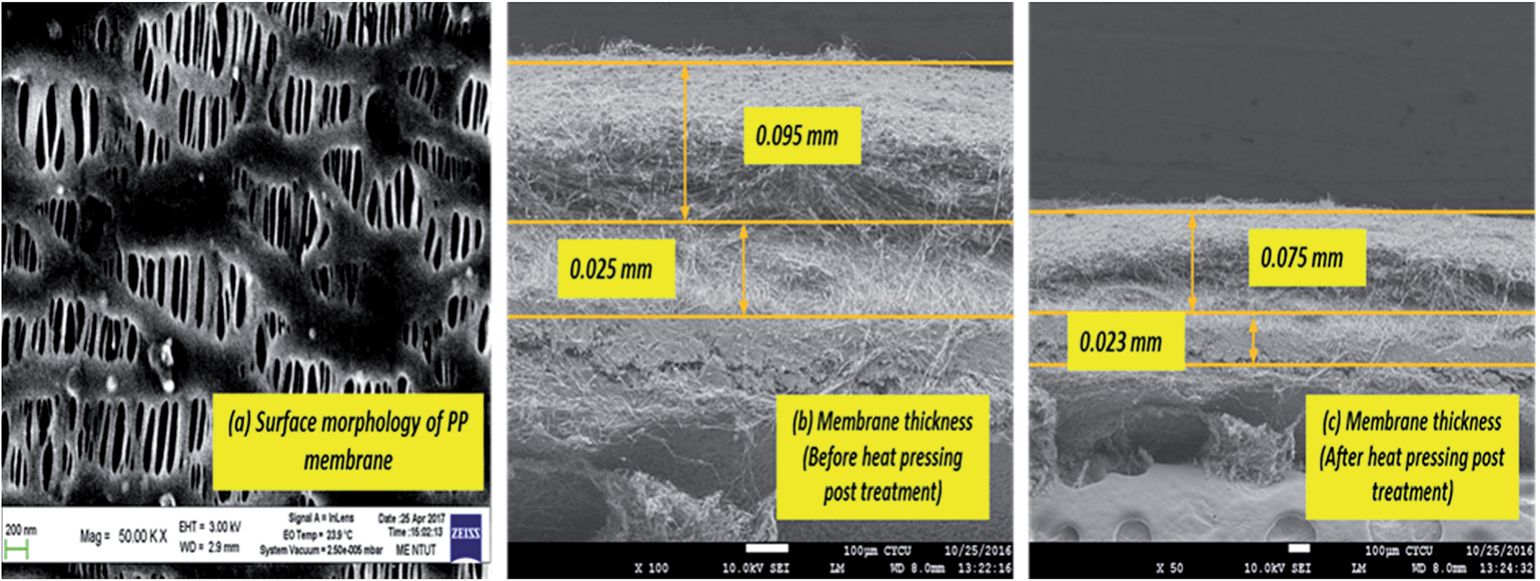
SEM micrograph: (a) surface morphology of the PP membrane. (b) Cross-sectional view of the PSF–CF/PP membrane before heat-pressing post-treatment. (c) Cross-sectional view of the PSF–CF/PP membrane after heat-pressing post-treatment.

Fig. 7 is a cross-sectional view of the PSF–CF/PP membrane, which also shows the morphology of the PP membrane. The SEM images indicate a significant decrease in membrane thickness after heat-pressing posttreatment.

#### 3.2.4 AFM analysis

**Fig. 8:**
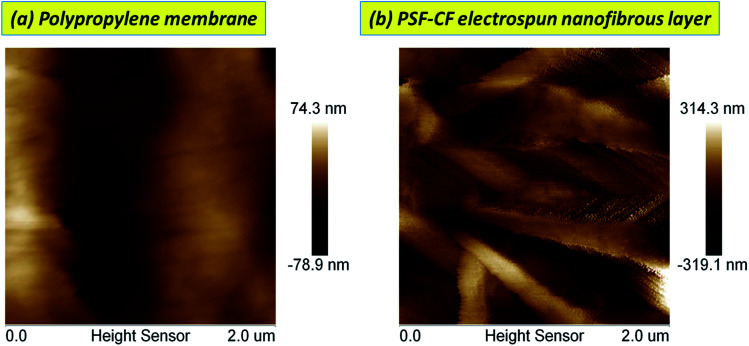
Two-dimensional AFM micrographs of membrane surfaces before and after modification by PSF–CF (dimensions: 2 μm × 2 μm).

Fig. 8 shows the 2D AFM morphology and indicates the corresponding surface roughness of the PP, PSF–PP, and PSF–CF/PP membranes. After the incorporation of Cera flava, the surface roughness increases considerably. Typically, a greater surface roughness results in a hierarchical surface structure in which air can be trapped to form a gas–liquid interface.^8*d*^ This leads to a more hydrophobic membrane surface. As mentioned earlier, Fig. 4 shows the contact angles of the following membranes; because Cera flava has a superhydrophobic composition, the hydrophobicity of the membrane surface is enhanced after the PSF–CF solution is electrospun onto it. Compared to the average contact angle of the PP membrane (92°), surface hydrophobicity increases with the concentration of PSF–CF on the PP membrane surface. The PSF–CF_(10%)_/PP membrane shows the maximum contact angle of 162°.

#### 3.2.5 Membrane surface analysis

Pore size and distribution are the most influential characteristics of membrane filtration quality.^24^ Typically, pore size and distribution not only influence the water flux but also have a high impact on rejection. Therefore, the surface of the membrane was analyzed using a Micromeritics N_2_ adsorption/desorption analyzer (ASAP 2020, USA). The data includes the BET surface area, Langmuir surface area, pore volume, and pore diameter.

**Fig. 9:**
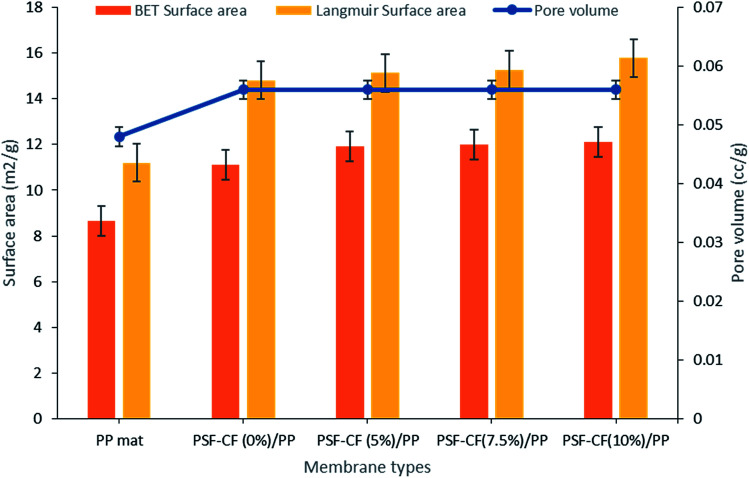
Surface area and pore volume analysis of various modified membranes [note: error bars are based on standard errors from three replicate tests].

The BET surface area indicates multilayer adsorption whereas the Langmuir surface area indicates monolayer adsorption.^25^ Initially, the BET and Langmuir surface areas seemed to increase after the modification of PP mat with PSF–CF, although with little difference in the pore volume. Fig. 9 indicates the surface area analysis along with the pore volume, which confirmed that almost all the modified membranes shows higher surface-area-to-volume ratio range. This effect can be seen in water flux as well which has been discussed in next part.

Table 4 shows the surface-area to volume ratio (considering BET surface area and Langmuir surface area). Interestingly, almost all the modified membranes possess higher surface area to volume ratio that indicates higher water flux range compared to PP mat.

**Table 4:** Analysis of surface area to pore volume ratio of various fabricated membrane

Membrane	BET surface area to volume ratio (m^2^ cc^−1^)	Langmuir surface area to volume ratio (m^2^ cc^−1^)
PP mat	180.41	233.33
PSF–CF_(0%)_/PP	198.21	264.2
PSF–CF_(5%)_/PP	212.67	271.41
PSF–CF_(7.5%)_/PP	214.28	272.5
PSF–CF_(10%)_/PP	214.47	281.78

The thickness of the PP membrane was found to be 0.020 mm, which is thin compared to other available commercial membranes. However, the membrane thickness was increased 3–4 times with the surface modification of electrospun PSF–CF nanofibers. In general, the membrane thickness may influence the water flux and decrease the thermal resistance (by reducing the heat efficiency or interface temperature difference) as the membrane becomes thinner.^26^ According to eqn (3), decreasing the thickness of the membrane also increases the sensible heat loss from the hot feed stream to the cold permeate stream, which leads to a decline of water flux because of decreased interfacial temperature differences (vapor pressure difference). Therefore, the membrane thickness must be optimized for efficient performance in MD processes.^20*a*,27^3
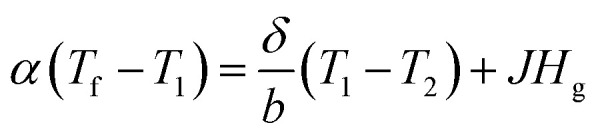
where, *T*_f_: feed temperature, *T*_1_: temperature drop across the feed stream, *T*_2_: temperature at the membrane surface in the cold permeate stream, *δ*: thermal conductivity of the membrane, *b*: membrane thickness, *α*: convective heat transfer coefficient on the feed stream, *J*: permeate water flux, and *H*_g_: enthalpy of the vapor.^20*a*^

Table 5 shows the comparative thicknesses as well as average contact angle of the different membranes before and after heat pressing treatment. Interestingly, the post heat treatment resulted in smoother surfaces that reduces average contact angle in the resultant membrane. Though the decrease in contact angle was found to be insignificant in this study, it can be suggested that the post heat-treatment has an impact on surface roughness as well as thickness of the membrane. The thickness of the surface-modified membrane was comparable to those of the commercially available membranes for MD applications (varying from 0.08 to 0.2 mm).

**Table 5:** Analysis of membrane thickness before and after heat pressing treatment^a^

Membrane types	Thickness before heat pressing treatment (mm)	Thickness after heat pressing treatment (mm)	Contact angle before heat pressing treatment (°)	Contact angle after heat pressing treatment (°)	Tensile strength after heat pressing treatment (MPa)
PP mat	0.025	0.023	92.1	90.5	33.1
PSF–CF_(0%)_/PP	0.121	0.092	131.7	130.1	34.6
PSF–CF_(5%)_/PP	0.122	0.096	152.6	150.7	34.6
PSF–CF_(7.5%)_/PP	0.120	0.095	160.1	158.9	35.1
PSF–CF_(10%)_/PP	0.122	0.095	163.9	162	35.2

a Note: tensile strength analysis has been conducted by utilizing an Instron mechanical tester at a tensile speed of 20 mm min^−1^.

**Fig. 10:**
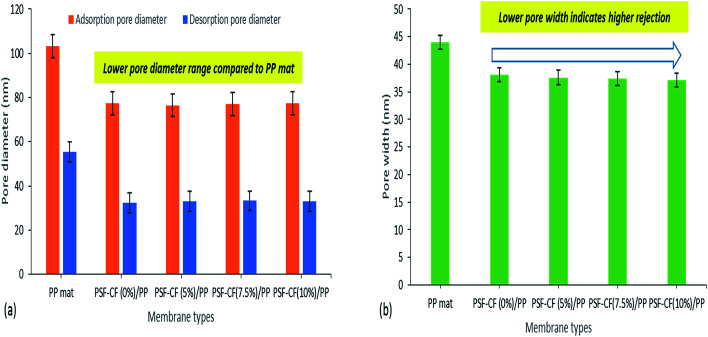
BJH adsorption/desorption average pore diameter and average pore width for various modified membranes [note: error bars were based on the standard errors of three replicate tests].

As reported earlier, capillary flow porometry (CFP) measures only the throat diameter of each through pore, one diameter per through pore is measured. Blind pores are not measured.^28^ Thus, in this study, BJH adsorption/desorption technique has been utilized to determine detailed information of membrane morphology. In addition to that, the pore width ranges from 20 nm to 35 nm for all the fabricated membranes and hence, BJH technique seems to be suitable for analysing the pore size. Even specific surface area, pore size distribution, total pore area and total pore volume can be easily measured by BJH adsorption/desorption technique. Hence, the average pore width and pore diameters of the PP and modified PSF–CF/PP membranes were analyzed using BJH adsorption/desorption techniques. Little difference was found in pore diameter when the concentration of PSF–CF was increased (Fig. 10). Therefore, the PSF–CF/PP membrane is expected to show a higher rejection percentage than the PP membrane because of the lower pore diameter range. In general, the salt rejection of a membrane design is dependent on both pore geometry and pore size; therefore, this study evaluated both the pore diameter and salt rejection. Smaller pore diameters clearly exhibited a higher rejection percentage. To support previous outcomes, the average pore widths of these membranes were analyzed, and PSF–CF/PP was found to possess a very small pore width (20 nm) compared with that of the PP membrane. Fig. 10 shows the pore width distribution based on different concentrations of PSF–CF on the PP membrane.

Typically, wetting can be directly analysed by contact angle. In general, smaller pore size, greater contact angle and surface tension increase the value of liquid entry pressure (LEP). The wetting of the membrane pores leads to reduced product quality; hence, it is advantageous to utilize membranes with high LEP value.^29^ Franken *et al.*^30^ has suggested a model to evaluate LEP value based on Cantor–Laplace equation^30^eqn (4):4
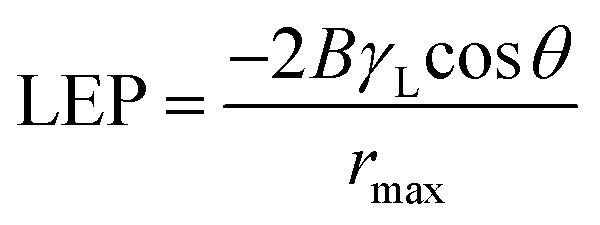
where LEP is the liquid entry pressure of pure water in Pa, *B* is a dimensionless geometrical factor that includes the irregularities of the pores (*B* = 1 for assumed cylindrical pores), *γ*_L_ is the liquid surface tension in N m^−1^ (in this case water at 25 °C, 0.07199 N m^−1^), cos *θ* is the contact angle in degree, *r*_max_ is the maximal pore (non-closed) radius in *m*.

**Fig. 11:**
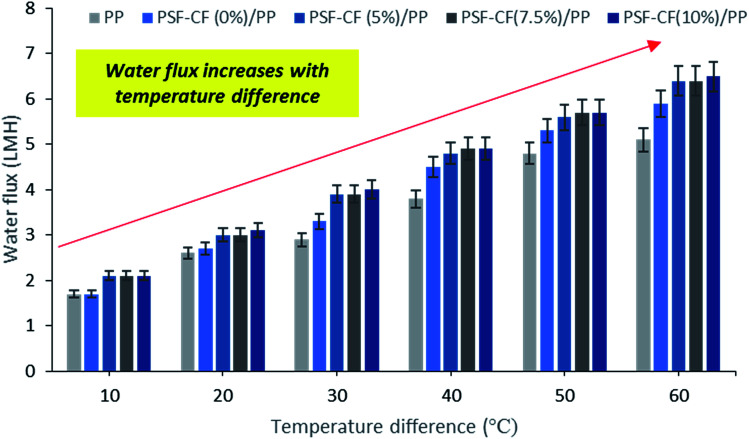
Effect of temperature difference on water flux in MD [note: feed solution = 30 g L^−1^ NaCl solution, time period = 1 h]. Error bars are based on standard errors from three replicate tests.

**Fig. 12:**
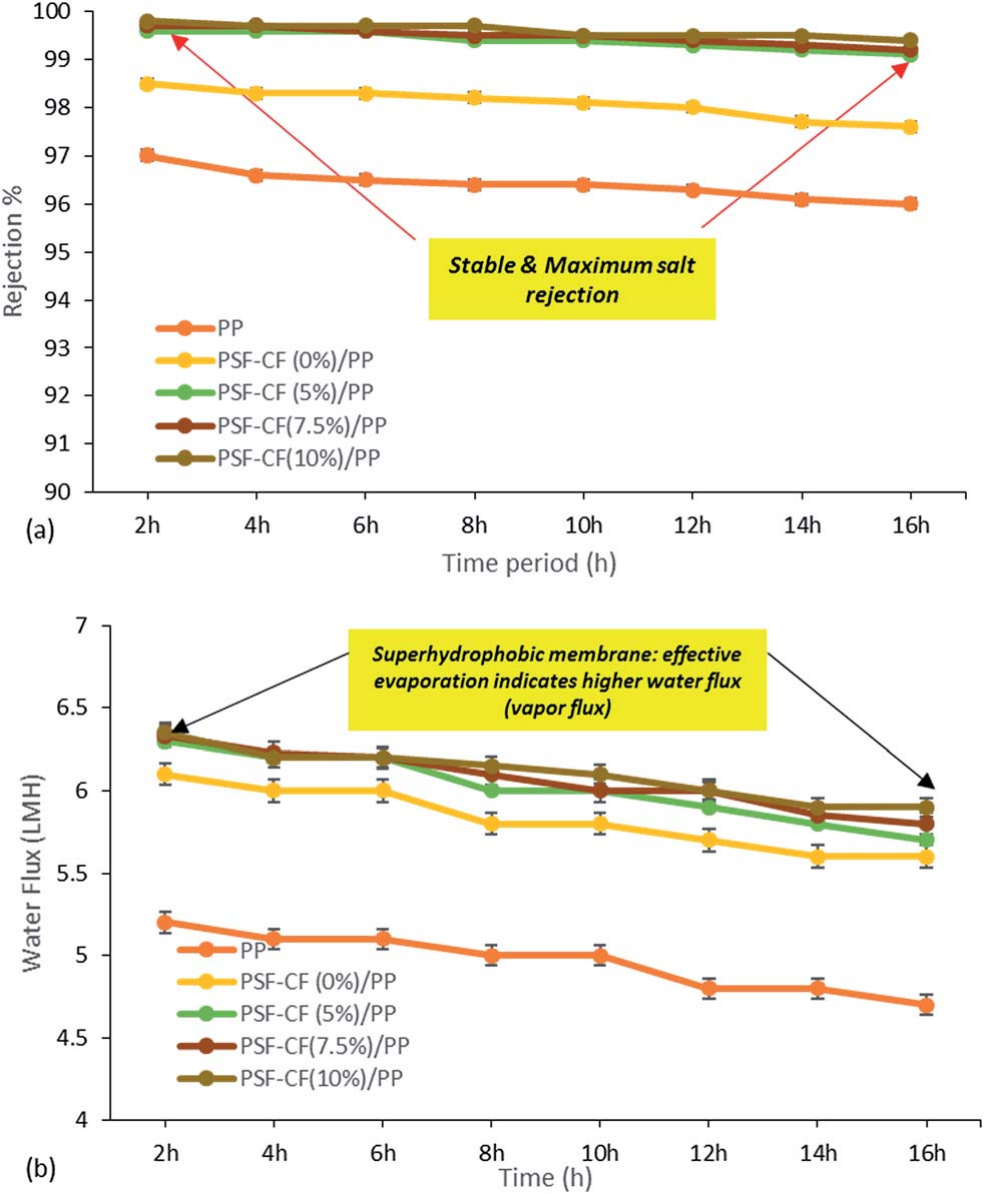
MD performance. (a) Effect of time interval on salt rejection. (b) Effect of time interval on permeate water flux of different fabricated membranes utilized in the MD system [note: feed solution = 30 g L^−1^ NaCl solution, temperature difference = 60 °C]. Error bars are based on standard errors from three replicate tests.

In addition to that, higher porosity is favoured for higher flux^31^Table 6 shows the LEP values that was calculated using the Cantor–Laplace equation whereas, the porosity of membrane was measured by BJH adsorption and desorption technique. Interestingly, the fabricated membranes PSF–CF/PP show higher degree of porosity than compared to PP mat which indirectly indicates higher water flux. This effect can be clearly seen in Fig. 11 and 12.

**Table 6:** Summary of membrane properties based on LEP and porosity [note: LEP was calculated by using Cantor–Laplace equation and porosity was measured by BJH adsorption/desorption technique; 1 Pa = 10^−5^ Bar]

Membrane Type	Average contact angle (°)	LEP (Bar)	Porosity (%)
PP mat	90.5°	0.22	71%
PSF–CF_(0%)_/PP	130.1°	4.12	80.1%
PSF–CF_(5%)_/PP	150.7°	4.58	80.2%
PSF–CF_(7.5%)_/PP	158.9°	4.79	80.2%
PSF–CF_(10%)_/PP	162.0°	4.97	81%

### 3.3 Membrane distillation application

The MD process was performed in the lab as indicated in Fig. 3. Aqueous NaCl solution with 30 g L^−1^ concentration was used as the feed solution. The temperature of the feed was controlled by a water bath, and varied between 30 °C and 80 °C. The temperature of the cooling side was constantly maintained at 20 °C by circulating a relatively large amount of water through an air cooling system. The effective membrane area measured 100 cm^2^ (10 cm × 10 cm). A mesh spacer was utilized in this MD process, working as a turbulence promoter that could reduce the thermal polarization on the feed stream of the membrane, resulting in higher mass transport.^32^ The salt concentration of the feed solution stream and the permeate water quality were measured by a professional series and a conductivity meter/TDS/DO (YSI Quatro, USA).

The results of the MD process demonstrate a sharp increase in water flux with an increasing temperature difference up to 80 °C (Δ*T* = 60 °C). The water flux varied from 4.5 to 6.4 LMH, which suggests stronger temperature dependence with the water flux. Thus, PSF–CF_(10%)_/PP shows maximum water flux for the highest surface-area-to-pore-volume ratio. Fig. 11 indicates an increase in water flux with an increase in temperature difference for the various membranes used in distillation.

Fig. 12 compares the water flux and salt rejection percentage over time. The MD process was continued for up to 16 hours for the various membranes to test their stability for long-term operation. The water flux and salt rejection declined slightly with increasing time duration. PSF–CF_(10%)_/PP possesses a maximum water flux of 6.4 LMH and rejection of 99.8%. The negligible water flux decline over time clearly indicates the higher stability and durability of the PSF sublayer on the PP membrane. Moreover, the interconnectivity of the PP membrane with the superhydrophobic layer (PSF–CF) is demonstrated by the stable water flux and salt rejection over 16 hours of operation. During the MD process, a stable water flux of 6.4 LMH was achieved, indicating the stability of the superhydrophobic/hydrophobic dual-layer composite membrane. Regarding surface area to pore volume ratio, the water flux of the PSF–CF/PP membrane was expected to exceed that of PP mat. Even, the superhydrophobic membrane composed of Cera flava indicates higher water flux compared to other membranes due to effective evaporation (higher vapor flux). In other words, the increased superhydrophobicity is responsible for higher and stable water flux while utilizing in MD application. Moreover, the salt rejection of all the modified membranes seems to be 99.5–99.8%, indicating higher permeate quality in the permeate stream as compared to the commercial PP mat which can be clearly identified in Fig. 12. The Cera flava based superhydrophobic modified membranes have indicated higher rejection as compared to hydrophobic membranes such as PSF/PP and PP mat. Thus, it can be concluded that, the superhydrophobicity has increased salt rejection in membrane distillation application due to higher wetting resistibility.

**Fig. 13:**
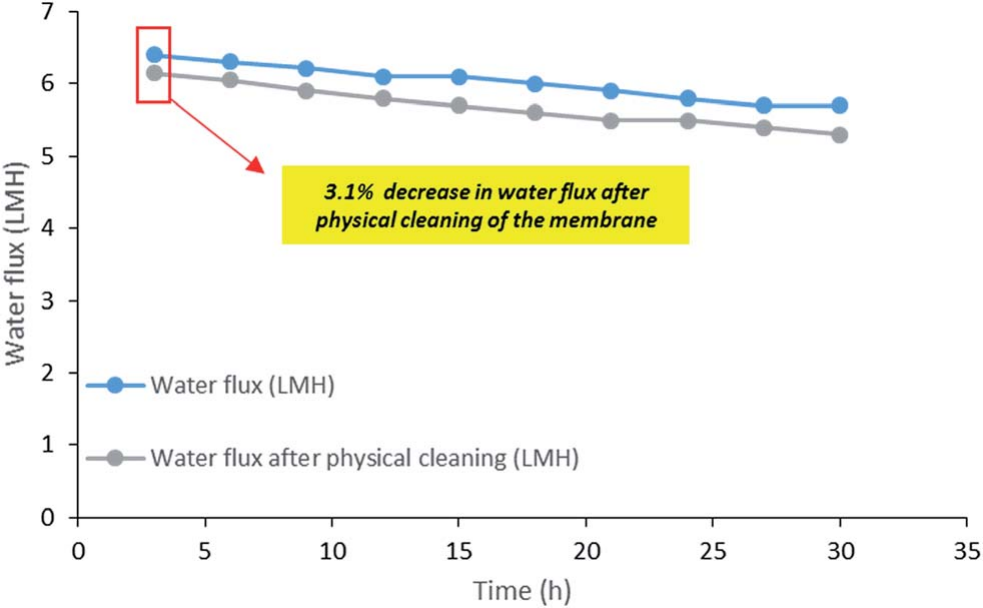
Long term performance analysis in terms of water flux decline: graphical representation of water flux and water flux after physical cleaning [note: membrane used: PSF–CF_(10%)_/PP, time: 30 h, feed stream: 30 g L^−1^, *T*_f_: 70 °C, *T*_p_: 20 °C].

The long term operation of PSF–CF_(10%)_/PP membrane was examined as it shows better efficiency compared to other membranes. Fig. 13 shows the reusability of the membrane which was analysed by evaluating the decrease in water flux after physical cleaning of the membrane. In this experiment, the same membrane was used for 30 h to analyse the long-term stability of the membrane. Interestingly, only 3.1% decrease in water flux initially was calculated. Thus, the decrease in water flux seems to be insignificant after reusing it for 30 h which directly indicates the self-cleaning property of this PSF–CF/PP superhydrophobic membrane.

## 4. Conclusion

In this study, electrospinning was used to generate a superhydrophobic nanofibrous nonwoven PSF layer on the surface of a hydrophobic PP membrane used for MD desalination. The results demonstrate that with a high voltage of 19–20 kV, a spinneret-to-collector distance of 12 cm, and a PSF solution concentration of 16% w/v incorporating Cera flava, the formation of superhydrophobic nanofibers can be achieved. The hybrid membrane consisting of 10 v/v% Cera flava (PSF–CF_(10%)_) possesses superhydrophobicity, with an average contact angle of approximately 162°. In addition, the membrane's morphology, contact angle, pore size distribution, BET surface area, and pore volume were thoroughly examined and compared with those of a standard PP membrane. The water flux and salt rejection percentage were also tested using 30 g L^−1^ of NaCl solution in the feed stream. Changes in the surface chemistry of the modified dual-layered PSF–CF/PP membranes resulted in a higher pore volume, producing twice the water flux of the PP membrane. Furthermore, this dual-layered membrane achieved 99.8% salt rejection. In conclusion, because the PSF–CF/PP membrane achieved an improved rejection percentage and stable water flux, PSF–CF is an ideal choice for modifying the surface of PP membranes for long-term MD operations.

## Conflicts of interest

The authors declare that they have no competing interests.

## Supplementary Material
